# Real-world data analysis of adjuvant capecitabine for triple-negative breast cancer after neoadjuvant chemotherapy

**DOI:** 10.3389/fonc.2025.1648272

**Published:** 2025-11-17

**Authors:** Ana Godoy-Ortíz, Gonzalo Lendínez-Sánchez, Manuel Zalabardo, Javier Pascual, Ana López-Pascual, Alexandra Cantero, Nuria Ribelles, Marcos Iglesias, Ester Villar, Bella Pajares, Tamara Díaz-Redondo, Maria Emilia Domínguez-Recio, Francisco Carabantes, Maria Jose Bermejo, Antonio Rueda-Domínguez, Emilio Alba, Alfonso Sánchez-Muñoz

**Affiliations:** 1 Department of Medical Oncology, Virgen de La Victoria University Hospital, Malaga, Spain; 2 The Biomedical Research Institute of Malaga (IBIMA-CIMES-UMA), Malaga, Spain; 3 Centro de Investigacion Biomedica en Red de Cáncer (CIBERONC-CB16/12/00481), Madrid, Spain; 4 Department of Medical Oncology, Hospital Regional Universitario de Malaga, Malaga, Spain; 5 Faculty of Medicine, University of Malaga, Malaga, Spain

**Keywords:** triple negative breast cancer, pathological complete response, adjuvant capecitabine, early breast cancer, CreateX, CIBOMA, BRCA1, BRCA2

## Abstract

**Purpose:**

Evaluate real-world outcomes in three cohorts of patients with early-stage triple-negative breast cancer (TNBC) treated with neoadjuvant chemotherapy (NAC): (1) patients who achieved pathological complete response (pCR); (2) patients without pCR who didn’t receive adjuvant chemotherapy; and (3) patients without pCR who received adjuvant capecitabine.

**Methods:**

Retrospective cross-sectional study from two hospitals in Málaga. Patients with TNBC received standard NAC followed by surgery. Between 2004 and 2015, patients not achieving pCR received no further systemic therapy. From 2015 onward, these patients were treated with adjuvant capecitabine. Kaplan–Meier and log-rank tests were used to compare disease-free survival (DFS) and overall survival (OS).

**Results:**

A total of 312 patients were included in the study. 133 achieved pCR, 84 patients didn’t achieve pCR and didn’t receive adjuvant capecitabine and 95 patients didn’t reach pCR and received adjuvant capecitabine. 89 patients experienced recurrence and 70 patients died. Patients who achieved pCR had a significantly higher DFS (HR 0.21 CI95% 0.12-0.36, p<0.0001) and higher overall survival (HR 0.27 CI95% 0.15-0.49, p<0.0001) compared to those who didn’t. Statistically significant differences in DFS and OS were observed among the three cohorts (DFS: p<0.00001; OS: p=0.00005). However, no statistically significant differences were found between cohorts 2 and 3 in terms of DFS (p=0.94) or OS (p=0.34).

**Conclusions:**

Patients who achieved pCR had better outcomes compared to those who didn’t. Among patients who didn’t achieve pCR, the addition of capecitabine didn’t result in significant improvements in DFS or OS compared to those who didn’t receive adjuvant treatment.

## 1 Introduction

Triple-negative breast cancer (TNBC) comprises a heterogeneous subgroup of tumors, accounting for approximately 15–20% of all breast cancers. TNBC is clinically defined by the absence of estrogen receptor (ER), progesterone receptor (PR), and human epidermal growth factor receptor 2 (HER2) amplification/overexpression. This breast cancer subtype exhibits a more aggressive natural history and worse disease-specific outcomes compared to other breast cancer subtypes. Standard treatment for early-stage TNBC has traditionally relied on anthracycline and taxane based chemotherapy, with or without platinum salts (1).

Neoadjuvant chemotherapy (NAC) has historically been used to downstage unresectable tumors, improving loco-regional control and increasing the rate of breast-conserving surgery. The neoadjuvant approach provides a valuable *in vivo* assessment of tumor biological sensitivity and drug efficacy. Patients who achieve pathological complete response (pCR) following NAC (between 30 - 40%), experience a significant improvement in prognosis, with disease-free survival (DFS) and overall survival (OS) comparable to that of patients with less aggressive tumors (2–4),,. However, TNBC patients with residual disease (RD) after chemotherapy face a high risk of relapse and mortality (2, 5), representing a critical unmet clinical need where post-neoadjuvant escalation therapies may still improve survival.

Following the CREATE-X trial, which demonstrated that adjuvant capecitabine improved both DFS and OS, its use as standard systemic therapy in unselected TNBC patients with RD after NAC has become standard therapy (6). The Finnish FinXX study evaluated adjuvant capecitabine in combination with standard adjuvant chemotherapy and, in an exploratory analysis of TNBC patients, reported a statistically significant improvement in recurrence-free survival (RFS, 7). However, the addition of capecitabine, whether concurrently or sequentially after standard adjuvant chemotherapy, has yielded controversial results (8, 9).

In this study, we assessed real-world outcomes in three cohorts of patients with early invasive TNBC treated with NAC based on anthracyclines-cyclophosphamide and/or taxanes, with or without carboplatin:

Cohort 1: Patients who achieved pCR and did not receive adjuvant therapy.

Cohort 2: Patients who did not achieve pCR and did not receive adjuvant chemotherapy or other systemic therapy.

Cohort 3: Patients who did not achieve pCR and received adjuvant chemotherapy with capecitabine.

## 2 Materials and methods

### 2.1 Patients and samples

We conducted a retrospective analysis of 312 patients with histologically confirmed invasive TNBC (ER-negative, PR-negative and Her2-negative). All patients received standard NAC. Within 4 to 6 weeks after the last chemotherapy cycle, patients underwent either modified radical mastectomy or conservative surgery with axillary lymph node dissection or sentinel lymph node biopsy according to standard surgical guidelines. Patients treated with breast-conserving surgery received adjuvant radiotherapy after surgery to the whole breast. For patients who underwent mastectomy, radiation therapy was administered to the chest wall and axillary region in patients who had an initial tumor > 5 cm, inflammatory breast cancer or had positive nodes before or after neoadjuvant chemotherapy.

**Figure 1:**
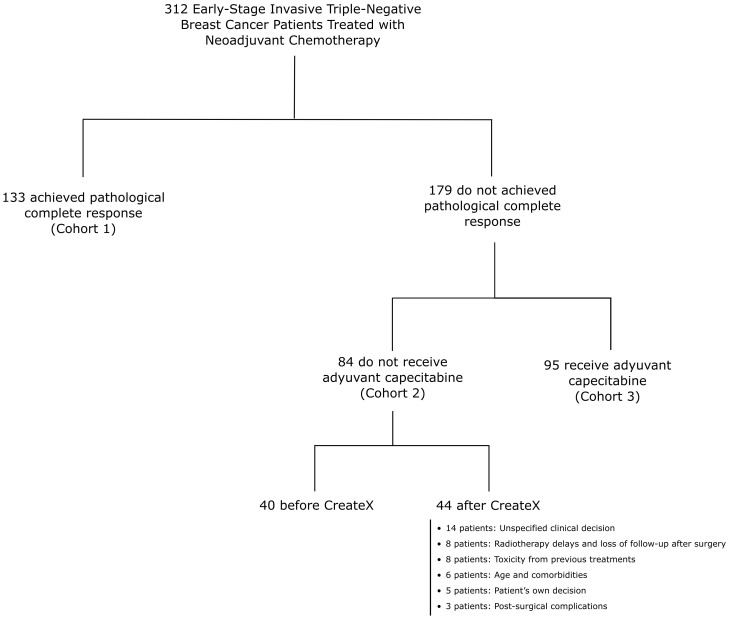
Flowchart of 312 patients with early-stage triple-negative breast cancer stratified by cohorts.

Patients who achieved a pCR did not receive chemotherapy adjuvant therapy or any other systemic therapy. From June 2004 to June 2015, patients who did not achieve pCR also did not receive adjuvant chemotherapy or other systemic treatments. However, since July 2015, patients with residual disease (RD) after NAC were administered adjuvant chemotherapy with oral capecitabine at a dose of 1250 mg/m² twice daily on days 1 to 14 of a 21-day cycle, for six to eight cycles, unless discontinued based on the clinical judgment of the oncologist, the patient’s decision, or any other reason as outlined in **Figure 1**. Patients who received Olaparib, pembrolizumab, or any adjuvant treatment other than capecitabine were excluded from the analysis. Patients were followed up at our institutions every six months, with annual mammographic screening to assess for breast cancer recurrence or upon presentation of any symptoms or signs suggestive of recurrence.

Tumor sample analyses were conducted on biopsy specimens obtained before neoadjuvant treatment. Immunohistochemical staining was performed at our hospital using the following antibodies: estrogen receptor (ER, Clone SP1), progesterone receptor (PR, Clone Y85), and human epidermal growth factor receptor 2 (HER2, HercepTest, DakoCytomation, Glostrup, Denmark).

All patient samples were classified as triple-negative based on immunohistochemistry (IHC), defined as ER-negative, PR-negative, and HER2-negative. ER and PR were considered negative if <1% of tumor cells showed positive staining. HER2 negativity was determined by either IHC scores of 0 or +1, or IHC + 2 with a negative fluorescence *in situ* hybridization (FISH) result.

Data on Ki-67 expression were collected both as a continuous variable and as a dichotomized variable, using a 50% cutoff. pCR was defined as the absence of invasive tumor cells in both the breast and lymph nodes (10). All patients provided written informed consent to receive treatment, and the study protocols were approved by the corresponding institutional ethics committees.

### 2.2 Statistical analysis methods

Continuous variables, including mean, median, measures of dispersion, standard deviation, interquartile range, and range, were summarized using measures of central tendency. Categorical variables were presented as frequencies and percentages.

Disease-free survival (DFS) was defined as the time from the date of surgery to either documented disease progression or death from any cause, whichever occurred first. Overall survival (OS) was defined as the time from the date of surgery to death from any cause. Kaplan–Meier curves and log-rank tests were used to compare DFS and OS among cohorts.

The impact of prognostic variables, including menopausal status, < 40 or ≥ 40 years, Ki-67 index ≤ 50% or > 50%, tumour size <5 cm or ≥5 cm, lymph node status previous to surgery, achieving a pCR after NAC and adjuvant capecitabine, was evaluated using Cox proportional hazards regression models.

Survival analysis stratified by germline status mutation in each cohort was estimated using Kaplan-Meier method.

All statistical analyses and figures were generated using SPSS statistical software, version 26 (IBM, Armonk, NY, USA).

Figures were modified using InkScape vector graphics editor software.

## 3 Results

Data from 312 women with early-stage TNBC treated with NAC between 2004 and 2023 were retrospectively collected. The median age at diagnosis was 48.5 years (range: 26-79) and 177 patients (56.7%) were premenopausal. Axillary lymphs node involvement at diagnosis was observed in 134 patients (42.9%) while 57 patients (18.3%) had a tumour size greater than 5 cm. Ki67 proliferation index was greater than 50% in 203 patients (65.1%). Germline mutation testing was conducted in 205 patients (65.7%), revealing 31 patients with a pathogenic BRCA1 mutation and 8 patients with a pathogenic BRCA2 mutation. However, these patients did not receive adjuvant treatment with Olaparib, as it was not yet approved at that time. Other mutations (detected in 12 patients) were ATM, PALB2, RAD51, CDKN2A, MUTYH and MSH6. The majority of patients (95%) received standard NAC, consisting of anthracyclines, cyclophosphamide, and taxanes, with or without carboplatin. Additional baseline clinical and pathological characteristics, stratified by cohort, are summarized in **Table 1**.

**Table 1:** Baseline clinical and pathological characteristics of the population.

	Total population	pCR	No pCR no adjuvant ChT	No pCR yes adjuvant Cht	P value
N	312	133 (42.6%)	84 (26.9%)	95 (30.5%)	
Age (median ± SD)	48.5 ± 11, 962				0.056
< 40	64 (20.5%)	35 (26.3%)	11 (13.1%)	18 (18.9%)	
≥ 40	248 (79.5%)	98 (73.7%)	73 (86.9%)	77 (81.1%)	
Menopausal status					**0.002**
Premenopause	177 (56.7%)	89 (66.9%)	36 (42.9%)	52 (54.7%)	
Postmenopause	135 (43.3%)	44 (33.1%)	48 (57.1%)	43 (45.3%)	
Histology					0.469
Ductal	279 (89.4%)	120 (43%)	73 (26.2%)	86 (30.8%)	
Lobular	4	1	2	1	
Medular	5	4	1	0	
Metaplasic	8	3	2	3	
Apocrine	4	0	1	3	
Other	12	5	5	2	
Tumoral Size					**0.003**
≤ 5 cm	255 (81.7%)	120 (90.2%)	62 (73.8%)	73 (76.8%)	
> 5cm	57 (18.3%)	13 (9.8%)	22 (26.2%)	22 (23.2%)	
N					**0.029**
N0	178 (57.1%)	87 (48.9%)	41 (23%)	50 (28.1%)	
N+	134 (42.9%)	46 (34.3%)	43 (32.1%)	45 (33.6%)	
Stage					0.079
I-II	254 (81.4%)	116 (87.2%)	65 (77.4%)	73 (76.8%)	
III	58 (18.6%)	17 (12.8%)	19 (22.6%)	22 (23.2%)	
Grade					0.162
G1	1 (0.3%)	0	0	1 (1.1%)	
G2	56 (18%)	18 (13.5%)	15 (17.9%)	23 (24.2%)	
G3	255 (81.7%)	115 (86.5%)	69 (82.1%)	71 (74.7%)	
Ki 67					**0.036**
≤ 50%	109 (34.9%)	36 (27.1%)	34 (40.5%)	39 (41.1%)	
> 50%	203 (65.1%)	97 (72.9%)	50 (59.5%)	56 (58.9%)	
Neoadjuvant ChT					0.533
DCT	289 (92.6%)	123 (92.5%)	75 (89.3%)	91 (95.8%)	
DCTC	9 (2.9%)	4 (3%)	2 (2.4%)	3 (3.1%)	
Other	14 (4.5%)	6 (4.5%)	7 (8.3%)	1 (1.1%)	
Surgery					0.295
Tumorectomy	203 (65.1%)	93 (69.9%)	53 (63.1%)	57 (60%)	
Radical Mastectomy	109 (34.9%)	40 (30.1%)	31 (36.9%)	38 (40%)	
Adjuvant radiotherapy					0.245
Yes	280 (89.7%)	115 (86.5%)	77 (91.7%)	88 (92.6%)	
No	32 (10.3%)	18 (13.5%)	7 (8.3%)	7 (7.4%)	
Germline mutation					
BRCA1	31 (9.9%)	20 (15%)	3 (3.6%)	8 (8.4%)	
BRCA2	8 (2.6%)	7 (5.3%)	1 (1.2%)	0	
NA	107 (34.3%)	34 (25.6%)	40 (47.6%)	33 (34.8%)	
Negative	154 (49.4%)	66 (49.6%)	40 (47.6%)	48 (50.5%)	
OTHERS	12 (3.8%)	6 (4.5%)	0	6 (6.3%)	

ChT, Chemotherapy; DCT, Doxorubicine-cyclophosphamide-taxane; DCTC, Doxorubicine-cyclophosphamide-taxane-carboplatin; NA, Not available; pCR, pathological complete response. *p* values from χ² tests evaluating differences in categorical variables between the three cohorts. Bold values indicate *p* < 0.05.

A pathological complete response was achieved by 133 patients (42.6%) who were categorized as Cohort 1. A total of 179 patients (57.4%) had RD. 84 patients (26.9%) did not receive adjuvant capecitabine and were categorized as Cohort 2. Notably, 44 patients in Cohort 2 did not receive adjuvant capecitabine treatment despite being diagnosed after the publication of the CreateX study results. The reasons for not receiving treatment are detailed in **Figure 1**. The remaining 95 patients (30.5%) received adjuvant capecitabine and were categorized as Cohort 3.

Median follow-up was of 48 months (range: 3-250; C1 = 59 months, C2 = 58 months; C3 = 39 months). A total of 89 patients (28.5%) experienced disease recurrence. Locoregional recurrence was observed in 24 patients (7.7%), while 63 patients (20.2%) developed distant metastases as their first site of progression. As of the time of this analysis (January 2025), 70 patients (23%) had died, including three patients who passed away without evidence of disease progression (due to sudden death, suicide, and small-cell lung cancer). Death, recurrence, and recurrence type stratified by cohort are summarized in **Table 2**.

**Table 2:** Recurrence and exitus stratified by cohort.

	Total population (n=312)	pCR (n=133)	No pCR no adjuvant ChT (n=84)	No pCR yes adjuvant Cht (n=95)
No recurrence	223	118	46	58
Recurrence	89	14	38	37
Exitus	70	13	33	24

**Figure 2:**
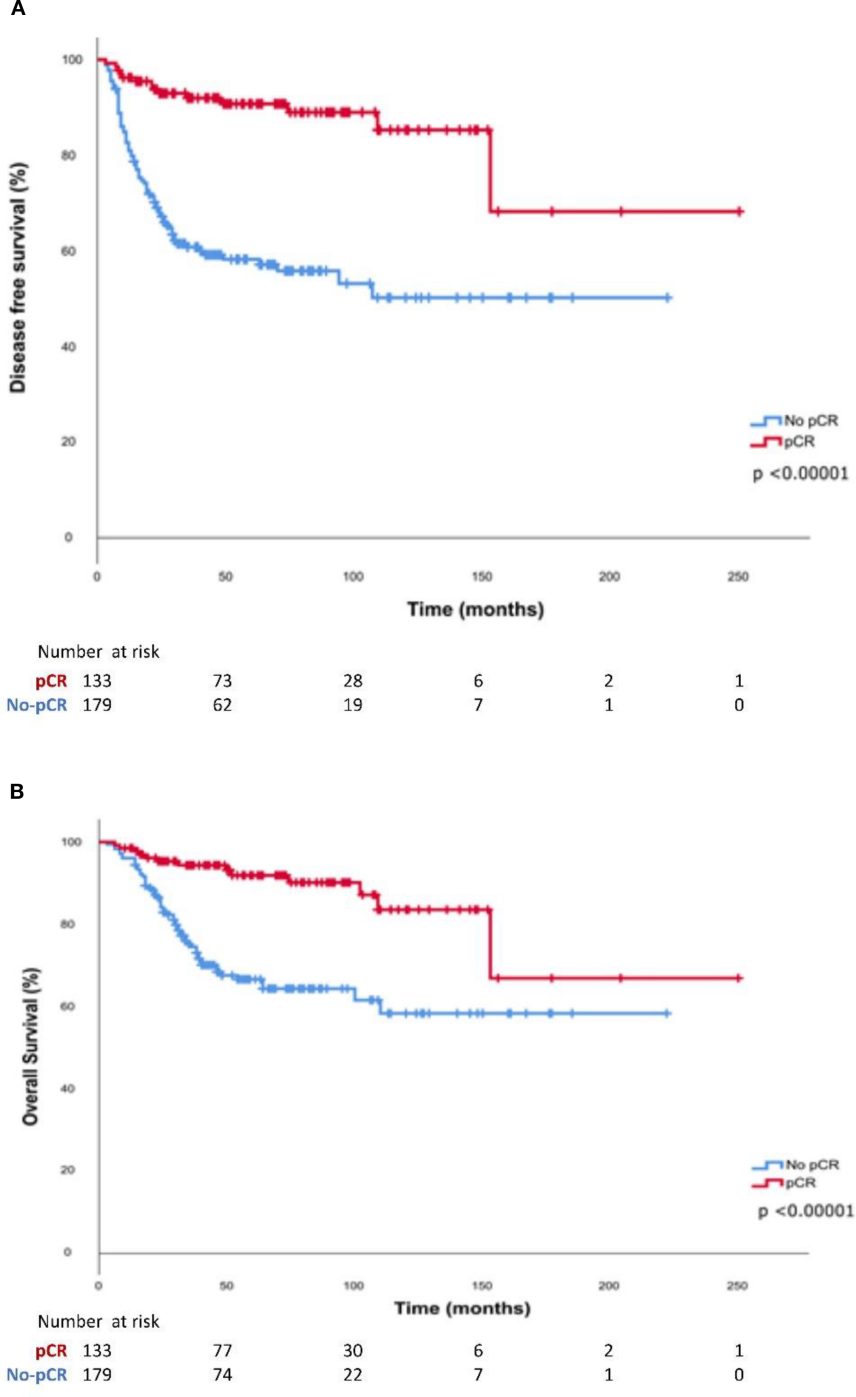
Kaplan-Meier curves of **(A)** Disease-free survival and **(B)** Overall survival according to pathological response after neoadjuvant treatment.

**Figure 3:**
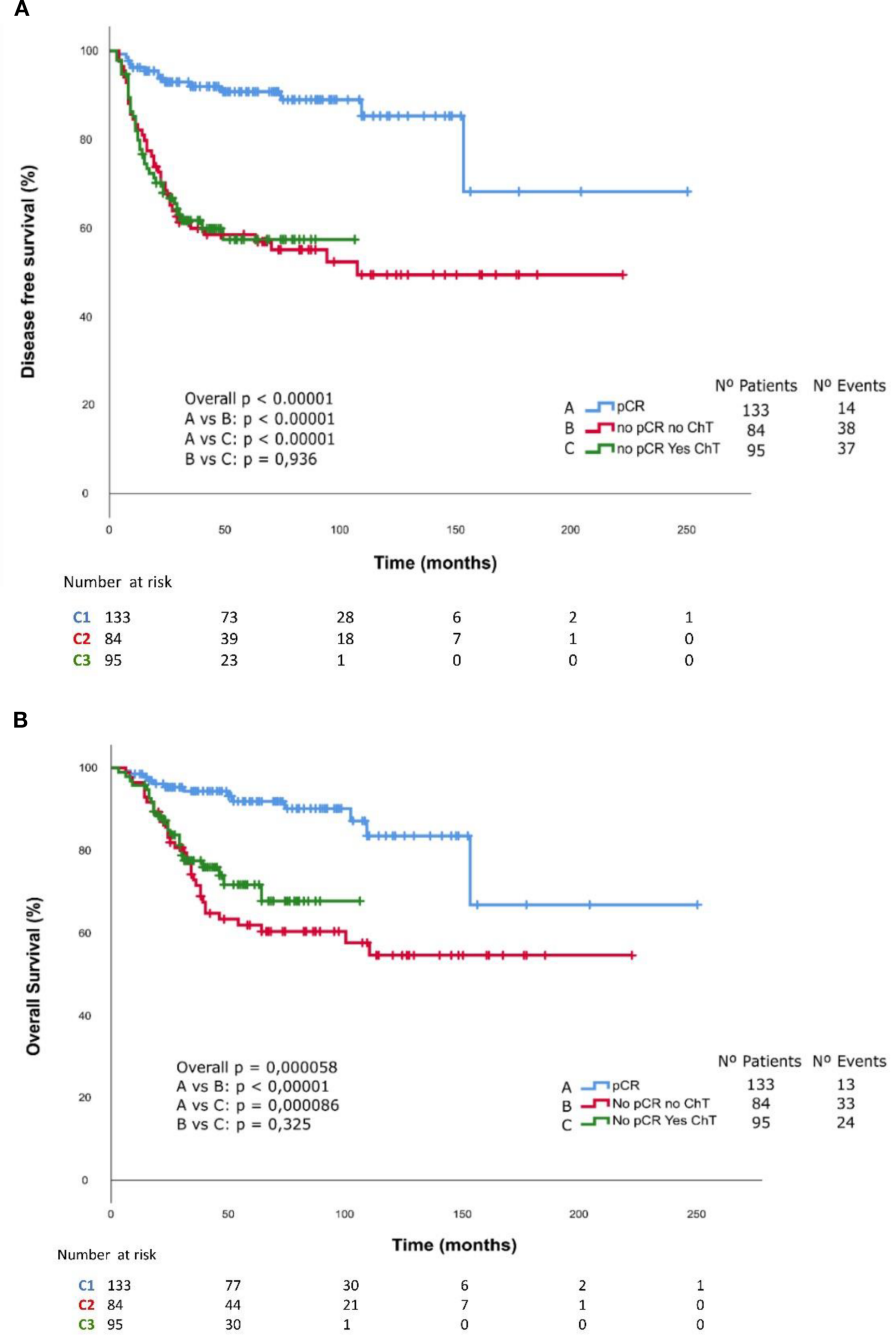
Kaplan-Meier curves of **(A)** Disease-free survival and **(B)** Overall survival stratified by cohort.

Patients who achieved a pCR had a significantly higher DFS (HR 0.21 CI95% 0.12-0.36, p<0.0001 and OS (HR 0.27 CI95% 0.15-0.49, p<0.0001) compared with those who did not achieve pCR (**Figures 2A, B**). Kaplan Meier curves and log-rank test for DFS and OS stratified by cohort are presented in **Figures 3A, B**. The median DFS and median OS were not reached in any of the three cohorts. Significant differences in DFS and OS were observed among the three cohorts according to the log-rank test (DFS: p < 0.00001; OS: p = 0.00005). However, no statistically significant differences were found between Cohort 2 and Cohort 3 in terms of DFS (p = 0.94) and OS (p = 0.34). The impact of multiple prognostic variables on DFS and OS — including menopausal status, < 40 or ≥ 40 years, Ki-67 index ≤ 50% or > 50%, tumour size <5 cm or ≥5 cm, lymph node status previous to surgery, achieving a pCR after NAC and adjuvant capecitabine — was evaluated using Cox regression analysis. Achieving a pCR was associated with a statistically significant lower risk of event in terms of both DFS (HR 0.25, 95% CI 0.133 – 0.467, p = 0.0001) and OS (HR 0.335, 95% CI 0.173– 0.646, p = 0.001). Additionally, Cox regression analysis revealed a significantly higher risk of events in terms of DFS (HR: 1.841, 95% CI: 1.201–2.821, p = 0.005) and OS (HR: 2.385, 95% CI: 1.445–3.939, p = 0.001) for patients with positive nodal status at diagnosis. Furthermore, patients with tumors >5 cm exhibited a significantly higher risk of events in terms of OS (HR: 1.742, 95% CI: 1.047–2.897, p = 0.033, **Supplementary Tables 1**, **2**). 

We conducted an exploratory analysis of DFS and OS stratified by germline mutation status within each cohort. Patients with undetermined germline status were excluded from this analysis. Among the 205 patients with available germline data, those carrying pathogenic mutations (n = 51) achieved significantly better outcomes in both DFS and OS compared with non-mutated patients (n = 154), with a DFS HR of 0.43 (95% CI = 0.20–0.96, p = 0.039) and an OS HR of 0.31 (95% CI = 0.11–0.88, p = 0.027). 

Six subgroups were subsequently defined according to the three main cohorts (1, 2, and 3) and germline mutation status (positive or negative). Patients in Cohort 1, who achieved a pCR, demonstrated the most favourable survival outcomes. Notably, none of the patients within this cohort who carried a germline mutation experienced disease recurrence or death during follow-up.

**Figure 4:**
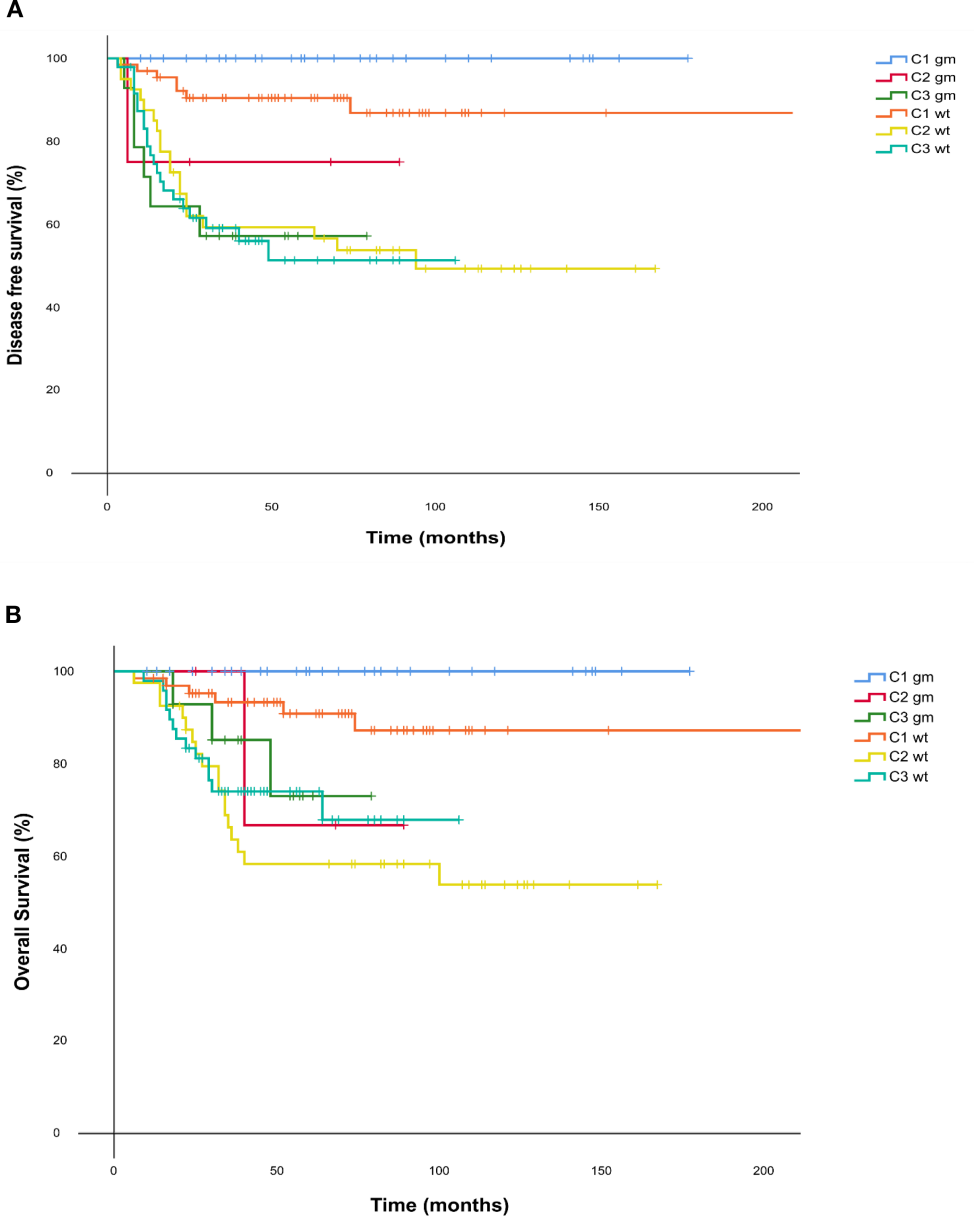
**(A)** Disease free survival and **(B)** Overall Survival Kaplan Meier curves for each cohort according to their mutational status. C1 = Cohort 1: pCR; C2 = Cohort 2: no pCR no adjuvant capecitabine; C3 = Cohort 3: no pCR yes adjuvant capecitabine. DFS, disease free survival; gm, germline mutated; OS, Overall survival; wt = wild type.

In contrast, patients in Cohorts 2 and 3 who did not achieve pCR showed poorer survival outcomes, irrespective of their germline mutation status or receipt of adjuvant capecitabine. Kaplan–Meier survival curves for DFS and OS according to germline status are presented in **Figures 4A, B**.

## 4 Discussion

In this retrospective study, we compared the outcome measured as DFS and OS of three cohorts of early TNBC patients treated with NAC: cohort 1 (patients who achieved a pCR); cohort 2 (not pCR and did not receive adjuvant chemotherapy); and cohort 3 (not pCR and adjuvant capecitabine). Our results demonstrated that patients who achieved a pCR (42.6%) had superior DFS and OS. However, Cohort 3 did not show an improved outcome compared to Cohort 2, suggesting the hypothesis that treatment with capecitabine in the absence of pCR after NAC may not provide a significant survival benefit in all patients with early TNBC.

The objective of precision medicine in TNBC implies that breast cancer treatment should be tailored based on the inherent risk of recurrence and individual sensitivity to different chemotherapies or alternative therapeutic approaches, such as targeted therapies or immunotherapy. First, it is essential to identify TNBC patients who require treatment beyond standard NAC. Two approaches may help in determining patients at the highest risk of recurrence and, therefore, in need of additional therapy. On an individual level, achieving a pCR is associated with improved survival (2, 5), whereas patients with residual disease (RD) face a higher risk of recurrence and mortality. Another strategy involves the use of highly sensitive diagnostic tests to detect and quantify minimal residual disease (MRD), such as circulating tumor cells (CTCs) or circulating tumor DNA (ctDNA), through blood-based “liquid biopsy” assays. The presence of these biomarkers during treatment has been linked to a less favorable prognosis in both early and advanced breast cancer (11, 12), although they do not yet have established predictive value.

Second, multiple clinical trials have explored the addition of novel treatment options to standard chemotherapy to improve patient outcomes. In this context, the phase III CREATE-X trial (6) aimed to identify a cohort of high-risk patients. The study included 910 women who had received standard NAC regimens and had RD after surgery. Patients were randomized to either no further therapy or six to eight cycles of sequential capecitabine. Capecitabine therapy improved 5-year DFS (70% vs. 56%) and OS (78.8% vs. 70.3%) in the subgroup of 286 women with TNBC. Based on these results, the use of adjuvant capecitabine in unselected TNBC patients with RD after NAC was established as the standard of care.

However, in the adjuvant setting, the addition of capecitabine—either concurrently, sequentially after standard chemotherapy, or as a metronomic regimen—has yielded controversial results in reducing the risk of recurrence. Furthermore, it substantially increases treatment toxicity without consistently improving OS (8, 13–16), or provides benefits only in certain subgroups of TNBC patients. For instance, in the phase III CIBOMA trial, only the non-basal TNBC subgroup (27% of patients, as expected) derived benefit from capecitabine, as observed in a retrospective and prespecified analysis (8). More recently, FOXC1, a transcriptional driver of cell plasticity and metastasis, was identified as a single biomarker assessed via standardized immunohistochemistry. This marker demonstrated the ability to identify basal-like breast cancer, further corroborating the lack of benefit of adjuvant capecitabine in basal TNBC subtyping according to PAM50 and IHC (17).

The phase III FinXX trial randomized patients to receive docetaxel with or without the addition of capecitabine, followed by epirubicin/cyclophosphamide. In the general population DFS and OS did not differ significantly between the groups after 10 years of follow up. Only in an exploratory subgroup analysis, a significant improvement in DFS and OS was observed in the TNBC subgroup, which comprised 202 patients (13% of the total population, 18). Furthermore, CREATE-X study included a preselected population with worse prognosis and a relative resistance to standard chemotherapy, conducted in an exclusively Asian population. This homogeneity may partially explain differences in capecitabine tolerance and efficacy (19). In contrast, studies based on real-world data and clinical experiences involving predominantly Caucasian populations, such as our study, provide valuable insights. In routine clinical practice, treatment strategies may differ from those applied in randomized controlled trials, making real-world studies a useful tool to assess the generalizability of trial results to broader, non-trial patient populations (20).

Preclinical models have supported the use of platinum agents in the basal TNBC subtype (21, 22). Addition of platinum agents to anthracycline and taxane consistently increased the pCR rates in clinical trials (23–25). Consequently, most oncologists have incorporated platinum agents into NAC regimens. However, these neoadjuvant trials were not powered to evaluate DFS or OS benefits. The results of the phase III EA1131 trial, which included patients with clinical stage II or III TNBC (basal vs. non-basal) and ≥1 cm residual disease in the breast post-NAC, demonstrated that adjuvant platinum agents did not improve outcomes and were associated with greater toxicity compared to capecitabine (26). Given that chemotherapy-based treatment may have reached the limit of its efficacy, novel therapeutic approaches are being explored for specific TNBC subgroups, particularly those with PD-L1-positive tumors or germline pathogenic BRCA mutations. However, only a fraction of these patients responds to immune checkpoint inhibitors (ICIs) or poly (ADP-ribose) polymerase (PARP) inhibitors, and even among responders, resistance and relapse frequently occur. The phase III Keynote-522 study demonstrated that the addition of pembrolizumab to NAC resulted in a statistically significant improvement in pCR rates, event-free survival, and OS (27). Another example is the phase III OlympiA trial, which included patients with HER2-negative early breast cancer with high-risk clinicopathological features and germline BRCA1 or BRCA2 pathogenic mutations who had received local treatment and neoadjuvant or adjuvant chemotherapy. The study compared one year of olaparib versus placebo, with most participants (81%) having TNBC. Patients who received olaparib had significantly improved distant DFS and OS, particularly among those who did not achieve a pCR. Notably, post-neoadjuvant capecitabine was not permitted in this trial, leaving the relative efficacy of olaparib compared to capecitabine in this setting unknown (28–30).

Subgroup analyses of the CREATE-X or GEICAM studies did not include germline BRCA mutation status, leaving the effect of adjuvant capecitabine in this population largely undefined. A correlative analysis of the GEICAM study, showed a decreased benefit from capecitabine in patients with basal-like tumors, which comprise the majority (~90%) of germline BRCA mutated breast cancer (6, 8, 31). Moreover, both a prespecified correlative analysis of the GEICAM/CIBOMA trial and findings from the EA1131 study suggest that patients with non-basal early-stage TNBC are more likely to derive benefit from adjuvant capecitabine (15, 26).

In our exploratory analysis, which grouped various germline mutations—predominantly BRCA1/2—we observed a trend toward improved outcomes among mutation carriers who achieved a pathological complete response (pCR), compared to non-carriers who also achieved a pCR.

Conversely, patients who failed to achieve a pCR exhibited poorer survival outcomes, regardless of germline mutation status or receipt of adjuvant capecitabine.

These findings should be interpreted with caution given the limited sample size, which may have restricted the ability to detect significant differences between subgroups. An increased sample size is necessary to determine whether our results are consistent with previously published findings in this setting.

Finally, our study has several limitations. As a retrospective study, establishing causal relationships is challenging due to the potential presence of unmeasured confounding factors. The lack of randomization may introduce biases that compromise the validity of the results, which are difficult to mitigate or correct. One potential limitation of our study is the temporal difference among the cohorts C1-C2 vs C3. This temporal gap may reflect improvements in diagnostic tools, surgical techniques and systemic treatments, which could have influenced patient outcomes. However, we attempted to minimize this potential bias by including clinicopathological and treatment-related variables in the multivariate model to adjust for possible confounders.

We believe that our findings provide relevant insights into the potential utility—or lack thereof—of adjuvant capecitabine in TNBC patients with residual disease (RD) after NAC in real-world settings. This is supported by the significant number of patients analyzed, the comparable clinico-pathological characteristics, and the similar NAC regimens used in Cohorts 2 and 3, along with an adequate follow-up period.

The visual differences observed in the Kaplan-Meier curves between Cohorts 2 and 3 in terms of OS may be attributed to differences in follow-up duration, as Cohort 3 had a shorter follow-up period than Cohort 2. With extended follow-up, the OS curve may more closely resemble the DFS curve, which are nearly overlapping.

In conclusion, adjuvant capecitabine may not provide a survival benefit for all TNBC patients with RD after NAC. A deeper understanding of distinct biological subtypes and molecular characteristics within RD will enable more personalized treatment decisions, optimizing therapeutic strategies for each patient subgroup while minimizing unnecessary toxicities.

## Data Availability

The raw data supporting the conclusions of this article will be made available by the authors, without undue reservation.
