# Supplementary material for: Real-world data analysis of adjuvant capecitabine for triple-negative breast cancer after neoadjuvant chemotherapy
Source: Front Oncol. 2025 Nov 17;15:1648272. doi: 10.3389/fonc.2025.1648272 (PMC12665565; doi:10.3389/fonc.2025.1648272)
Supplement: Supplementary file 1 [file Table1.docx]

Supplementary Table 1: Multivariate Cox Regression Analysis of Clinicopathological Variables for Disease-Free Survival

| **Variable** | **Hazard ratio (95% CI)** | **P value** |
| --- | --- | --- |
| < 40y vs ≥ 40y | 1.321 (0.674 – 2.589) | 0.418 |
| Postmenopause vs Premenopause | 0.898 (0.562 – 1.436) | 0.653 |
| > 5 cm vs ≤ 5cm | 1.397 (0.872 – 2.238) | 0.164 |
| **N+ vs N0** | **1.841 (****1.201 – 2.821)** | **0.005** |
| Ki67 >50% vs ≤50% | 1.071 (0.691 – 1.662) | 0.758 |
| **pCR vs Non pCR** | **0.25 (****0.133 – 0.467)** | **<0.001** |
| Adjuvant capecitabine | 1.048 (0.659 – 1.665) | 0.844 |

Supplementary Table 2: Multivariate Cox regression analysis of Clinicopathological variables for Overall Survival

| **Variable** | **Hazard ratio (95% CI)** | **P value** |
| --- | --- | --- |
| < 40y vs ≥ 40y | 1.673 (713 – 3.927) | 0.237 |
| Postmenopause vs Premenopause | 0.737 (0.433 – 1.254) | 0.261 |
| **> 5 cm vs ≤ 5cm** | **1.742 (****1.047 – 2.897)** | **0.033** |
| **N+ vs N0** | **2.385 (****1.445 – 3.939)** | **0.001** |
| Ki67 >50% vs ≤50% | 1.115 (0.678 – 1.832) | 0.668 |
| **pCR vs Non pCR** | **0.335 (****0.173– 0.646)** | **0.001** |
| Adjuvant capecitabine | 0.835 (0.485 – 1.437) | 0.516 |
